# TS-EUROTRAIN: A European-Wide Investigation and Training Network on the Etiology and Pathophysiology of Gilles de la Tourette Syndrome

**DOI:** 10.3389/fnins.2016.00384

**Published:** 2016-08-23

**Authors:** Natalie J. Forde, Ahmad S. Kanaan, Joanna Widomska, Shanmukha S. Padmanabhuni, Ester Nespoli, John Alexander, Juan I. Rodriguez Arranz, Siyan Fan, Rayan Houssari, Muhammad S. Nawaz, Francesca Rizzo, Luca Pagliaroli, Nuno R. Zilhäo, Tamas Aranyi, Csaba Barta, Tobias M. Boeckers, Dorret I. Boomsma, Wim R. Buisman, Jan K. Buitelaar, Danielle Cath, Andrea Dietrich, Nicole Driessen, Petros Drineas, Michelle Dunlap, Sarah Gerasch, Jeffrey Glennon, Bastian Hengerer, Odile A. van den Heuvel, Cathrine Jespersgaard, Harald E. Möller, Kirsten R. Müller-Vahl, Thaïra J. C. Openneer, Geert Poelmans, Petra J. W. Pouwels, Jeremiah M. Scharf, Hreinn Stefansson, Zeynep Tümer, Dick J. Veltman, Ysbrand D. van der Werf, Pieter J. Hoekstra, Andrea Ludolph, Peristera Paschou

**Affiliations:** ^1^Department of Psychiatry, University of Groningen, University Medical Center GroningenGroningen, Netherlands; ^2^Department of Cognitive Neuroscience, Donders Institute for Brain, Cognition and Behaviour, Radboud University Medical CenterNijmegen, Netherlands; ^3^Clinic of Psychiatry, Social Psychiatry and Psychotherapy, Hannover Medical SchoolHannover, Germany; ^4^Max Planck Institute for Human Cognitive and Brain SciencesLeipzig, Germany; ^5^Department of Molecular Biology and Genetics, Democritus University of ThraceAlexandropoulos, Greece; ^6^Boehringer Ingelheim Pharma GmbH & Co. KG, CNS ResearchBiberach an der Riss, Germany; ^7^Department of Child and Adolescent Psychiatry, University of UlmUlm, Germany; ^8^Department of Clinical Genetics, Applied Human Molecular Genetics, Kennedy Center, Copenhagen University HospitalRigshospitalet, Denmark; ^9^Department of Clinical and health Psychology, Utrecht UniversityUtrecht, Netherlands; ^10^Department of Psychiatry, VU University Medical CenterAmsterdam, Netherlands; ^11^Department of Anatomy and Neurosciences, VU University Medical CenterAmsterdam, Netherlands; ^12^deCODE Genetics/AmgenReykjavik, Iceland; ^13^Faculty of Medicine, University of IcelandReykjavik, Iceland; ^14^Institute for Anatomy and Cell Biology, Ulm UniversityUlm, Germany; ^15^Institute of Medical Chemistry, Molecular Biology and Pathobiochemistry, Semmelweis UniversityBudapest, Hungary; ^16^Research Centre for Natural Sciences, Institute of Enzymology, Hungarian Academy of SciencesBudapest, Hungary; ^17^Department of Biological Psychology, VU UniversityAmsterdam, Netherlands; ^18^Université d'Angers, BNMI (Institut national de la santé et de la recherche médicale 1083 / Centre National de la Recherche Scientifique 6214)Angers, France; ^19^EMGO+ Institute for Health and Care Research, VU University Medical CentreAmsterdam, Netherlands; ^20^Stichting Gilles de la TouretteRhoon, Netherlands; ^21^Karakter Child and Adolescent Psychiatry, University CentreNijmegen, Netherlands; ^22^Rensselaer Polytechnic InstituteTroy, NY, USA; ^23^Tourette-Gesellschaft Deutschland e.V.Hannover, Germany; ^24^Department of Human Genetics, Radboud University Medical CenterNijmegen, Netherlands; ^25^Department of Molecular Animal Physiology, Donders Institute for Brain, Cognition and Behaviour, Radboud Institute for Molecular Life Sciences, Radboud UniversityNijmegen, Netherlands; ^26^Department of Physics and Medical Technology, VU University Medical CenterAmsterdam, Netherlands; ^27^Psychiatric and Neurodevelopmental Genetics Unit, Departments of Psychiatry and Neurology, Center for Human Genetic Research, Harvard Medical School, Massachusetts General HospitalBoston, MA, USA; ^28^Netherlands Institute for NeuroscienceAmsterdam, Netherlands

**Keywords:** Initial Training Network, Gilles de la Tourette Syndrome, tourette disorder, etiology, genetics, neuroimaging, animal models

## Abstract

Gilles de la Tourette Syndrome (GTS) is characterized by the presence of multiple motor and phonic tics with a fluctuating course of intensity, frequency, and severity. Up to 90% of patients with GTS present with comorbid conditions, most commonly attention-deficit/hyperactivity disorder (ADHD), and obsessive-compulsive disorder (OCD), thus providing an excellent model for the exploration of shared etiology across disorders. TS-EUROTRAIN (FP7-PEOPLE-2012-ITN, Grant Agr.No. 316978) is a Marie Curie Initial Training Network (http://ts-eurotrain.eu) that aims to elucidate the complex etiology of the onset and clinical course of GTS, investigate the neurobiological underpinnings of GTS and related disorders, translate research findings into clinical applications, and establish a pan-European infrastructure for the study of GTS. This includes the challenges of (i) assembling a large genetic database for the evaluation of the genetic architecture with high statistical power; (ii) exploring the role of gene-environment interactions including the effects of epigenetic phenomena; (iii) employing endophenotype-based approaches to understand the shared etiology between GTS, OCD, and ADHD; (iv) establishing a developmental animal model for GTS; (v) gaining new insights into the neurobiological mechanisms of GTS via cross-sectional and longitudinal neuroimaging studies; and (vi) partaking in outreach activities including the dissemination of scientific knowledge about GTS to the public. Fifteen partners from academia and industry and 12 PhD candidates pursue the project. Here, we aim to share the design of an interdisciplinary project, showcasing the potential of large-scale collaborative efforts in the field of GTS. Our ultimate aims are to elucidate the complex etiology and neurobiological underpinnings of GTS, translate research findings into clinical applications, and establish Pan-European infrastructure for the study of GTS and associated disorders.

## Introduction

Gilles de la Tourette Syndrome (GTS) is a frequent disorder (0.4–1%; Robertson, 2008, 2015b), characterized by multiple motor and phonic tics and high comorbidity with attention-deficit/hyperactivity disorder (ADHD; 50%) and obsessive-compulsive disorder (OCD; 20–60%) (Leckman et al., 1998; Robertson, 2000; Bloch and Leckman, 2009; Debes et al., 2010; American Psychiatric Association, 2013; Hirschtritt et al., 2015). The need to overcome fragmentation and accelerate research into the etiology of GTS and its related conditions has motivated the establishment of TS-EUROTRAIN (http://ts-eurotrain.eu), a Marie Curie Initial Training Network (ITN, 2012–2016) that focuses on the investigation of the genetic etiology and pathophysiology of GTS while aiming to translate findings into clinical research. The network spans 13 academic and two industrial partners as well as two patient groups. Twelve individual, yet complementary, PhD projects interact to form a comprehensive study of GTS and comorbidities from genetics, and epigenetics through to physiology, brain anatomy, and function. These projects are all currently underway and can roughly be divided into three groups by their main approach; genetic (and epigenetic), animal models, and human neuroimaging, respectively. Research into the neurobiology of GTS stands at the precipice of discovery thanks to collaborative efforts (Georgitsi et al., 2016). With this report, we would like to share our efforts as an example of how, taking advantage of expertise across different disciplines, and resources across the GTS scientific and patient community we aimed to build a project that would achieve goals beyond and above the reach of individual labs. At the same time we provide an overview of some of the largest-scale projects aiming to understand the etiology of GTS. These projects may be expected to impact the field considerably in the coming years.

## Genetics, epigenetics, and gene expression

**Figure 1:**
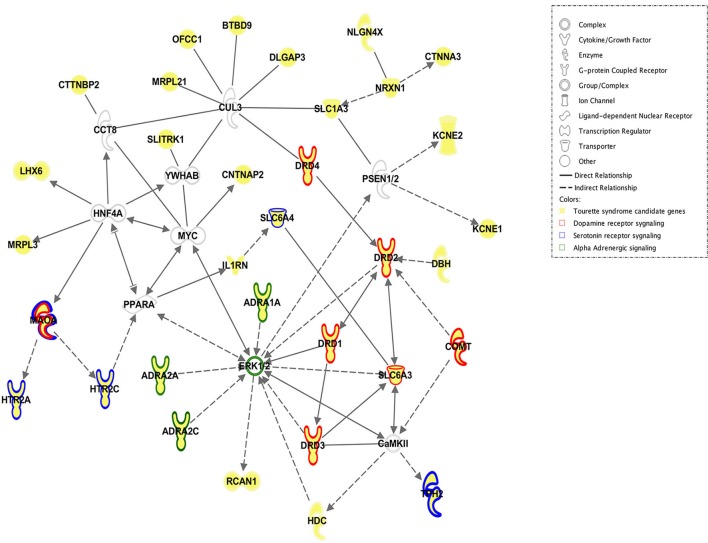
**Network of reported candidate genes associated with GTS**. This image was produced (by JW) with Ingenuity pathway analysis software and shows how the proteins encoded by the candidate genes reported to be associated with GTS are linked with each other. Please see legend for description of what each symbol and color represents. ADRA1A, adrenoceptor alpha 1A; ADRA2A, adrenoceptor alpha 2A; ADRA2C, adrenoceptor alpha 2C; BTBD9, BTB (POZ) domain containing 9; CaMKII, calcium/calmodulin-dependent protein kinase II; CCT8, chaperonin containing TCP1, subunit 8 (theta); CNTNAP2, contactin-associated protein-like 2; COMT, catechol-O-methyltransferase; CTNNA3, catenin (cadherin-associated protein), alpha 3; CTTNBP2, contactin binding protein 2; CUL3, cullin 3; DBH, dopamine beta-hydroxylase (dopamine beta-monooxygenase); DLGAP3, discs; large (Drosophila) homolog-associated protein 3; DRD1, dopamine receptor D1, DRD2, dopamine receptor D2, DRD3, dopamine receptor D3, DRD4, dopamine receptor D4, ERK 1/2, extracellular signal-regulated kinases 1/2; HDC, histidine decarboxylase, HNF4A, hepatocyte nuclear factor 4 alpha; HTR2A, 5-hydroxytryptamine (serotonin) receptor 2A; G protein-coupled; HTR2C, 5-hydroxytryptamine (serotonin) receptor 2C; G protein-coupled; IL1RN, interleukin 1 receptor antagonist; KCNE1, potassium channel voltage gated subfamily E regulatory beta subunit 1; KCNE2, potassium channel, voltage gated subfamily E regulatory beta subunit 2; LHX6, LIM homeobox 6; MAOA, monoamine oxidase A; MRPL21, mitochondrial ribosomal protein L21; MRPL3, mitochondrial ribosomal protein L3; MYC, v-myc avian myelocytomatosis viral oncogene homolog; NLGN4X, neuroligin 4, X-linked; NRXN1, neurexin 1; OFCC1, orofacial cleft 1 candidate 1; PPARA, peroxisome proliferator-activated receptor alpha; RCAN1, regulator of calcineurin 1; PSEN1/2, presenilin 1/2; SLC1A3, solute carrier family 1 (glial high affinity glutamate transporter), member 3; SLC6A3, solute carrier family 6 (neurotransmitter transporter); member 3; SLC6A4, solute carrier family 6 (neurotransmitter transporter), member 4, SLITRK1, SLIT, and NTRK-like family, member 1; TPH2, tryptophan hydroxylase 2; YWHAB, tyrosine 3-monooxygenase/tryptophan 5-monooxygenase activation protein, beta.

The first Genome-wide Association Study (GWAS) to investigate the role of single nucleotide polymorphisms (SNPs) in GTS did not manage to identify SNPs that meet the genome-wide significance level for association to GTS, however, four additional GWAS for GTS are currently underway [coordinated by the Tourette Association International Consortium for Genetics (TSAICG), European Multicentre Tics in Children Studies (EMTICS), Netherlands twin register (NTR) and deCODE] and the future meta-analysis of these datasets is expected to provide important insights into the etiology of the disorder (Figure 1; Paschou, 2013; Scharf et al., 2013). Furthermore, in recent years, four independent GTS cohorts have been examined, studying the role of Copy Number Variants (CNVs) in GTS (Sundaram et al., 2010; Fernandez et al., 2012; Nag et al., 2013; McGrath et al., 2014). Regarding gene expression investigations, so far, most studies were carried out on samples of small number (Tang et al., 2005; Lit et al., 2007, 2009; Liao et al., 2010; Tian et al., 2011a,b, 2012; Gunther et al., 2012; Gomez et al., 2014; Lennington et al., 2016) and need to be verified in large GTS cohorts. On the other hand, studies on the epigenetics of GTS (such as DNA methylation, histone modification, and micro-RNA (miRNA) alteration Goldberg et al., 2007; Pagliaroli et al., 2016) remain scarce (Abelson et al., 2005; Delgado et al., 2014) and in fact, the first ever epigenome-wide study for GTS was only recently published through TS-EUROTRAIN efforts (Zilhão et al., 2015). We address the whole spectrum of GTS genetics from various angles; genetic, epigenetic, gene expression, and their interaction with environmental factors.

### Project 1 genome-wide search for genes conferring risk of GTS (Muhammad Sulaman Nawaz and Hreinn Steffanson, decode genetics)

This project makes use of the extensive Icelandic population genotyping done by deCODE genetics. Approximately one third of the population (100,000) has been genotyped into which 20,000,000 SNPs from the Icelandic sequencing project have been imputed. Tasks include (i) a genome-wide search for genetic variants conferring risk of GTS. This consists of a search for common and rare variants in more than 500 chip typed subjects diagnosed with GTS, (ii) a genome wide search for CNVs associated with GTS, (iii) a test for association of identified variants with phenotypic measures as well as performance on neuropsychological tests, (iv) an investigation of how implicated variants may lead to alteration of gene-expression pathways through analysis of already generated expression cohorts.

### Project 2 investigation of the role of CNVs as genetic susceptibility factors involved in the pathogenesis of GTS and co-morbid disorders (Rayan Houssari, Juan Ignacio Rodriguez Arranz, Mehar Arumilli, and Zeynep Tümer, Kennedy Center, Copenhagen University Hospital, Rigshospitalet)

The aim of this project is to untangle novel molecular genetic mechanisms underlying GTS and related disorders, by using bioinformatic network analysis of CNVs combined with phenotype data of 261 GTS-patients residing in Denmark. All the patients were assessed by experienced clinicians at the Tourette Clinic, Copenhagen University Hospital for GTS, OCD, and ADHD using validated diagnostic instruments (Mol Debes et al., 2008). Furthermore, information about other family members was collected through interviews revealing approximately 77% of the families to be multiplex with at least two family members affected by GTS or one of the common comorbidities. A biobank consisting of cell-lines, DNA, RNA, and serum has been established. All the patients have been screened using the Affymetrix CytoScan HD chromosome microarray platform with more than 2.6 million copy number markers and the bioinformatic data analysis is under way. This study, in collaboration with other members of the network, has already enabled identification of the *AADAC* gene as a susceptibility factor for GTS when deleted (Bertelsen et al., 2016).

### Project 3 gene-environment interactions defining the onset and clinical course of tics and obsessive-compulsive symptoms (Shanmukha Sampath Padmanabhuni and Peristera Paschou, Democritus University of Thrace)

The aim of this project is to investigate the interaction between genetic and environmental factors that may lead to the onset of tics. Following a systems biology approach information from multiple sources are integrated; including genome-wide genotyping, gene expression patterns, epigenetics, and longitudinal clinical observations. Through collaboration with the FP7-HEALTH project EMTICS, a special focus is placed on group A streptococcal infections and stress as a possible trigger for tic onset. EMTICS also offers us access to genome-wide genotype data of 1000 patients (followed up on a monthly basis for 12 months) as well as gene expression data on 200 GTS patients that are followed up for tic exacerbation and remission in an attempt to correlate with environmental factors. Gene-expression and correlation with environmental triggers is also investigated in a cohort of first degree relatives of patients with GTS that develop tic symptomatology within a 3-year follow-up period. Furthermore, the first ever epigenome-wide association study for tics, analysing data from the NTR, has been carried out [55]. This study comprised the largest epigenetic data collection so far undertaken (411,469 autosomal methylation sites, assessed in 1678 individuals). Although no site reached genomewide significance, the top hits include several genes, and regions previously associated with neurological disorders and warrant further investigation (Zilhão et al., 2015).

### Project 4 epigenetic and functional characterization of proposed genetic variants and regions implicated in the pathogenesis of GTS and related phenotypes (Luca Pagliaroli and Csaba Barta, Semmelweis University)

The aim of this project is to shed light on the main epigenetic mechanisms, such as DNA methylation, histone modification and miRNA, and their possible role in GTS. Tasks include (i) the study of candidate miRNAs which are predicted to be in the control of tissue-specific gene expression by *in vivo* target validation of *in silico* proposed miRNA target genes, (ii) screening of cell lines and GTS animal models treated with dopaminergic and glutamatergic modulating compounds for epigenetic regulatory markers, (iii) investigation of brain tissue samples from treated and untreated animal models developed within the TS-EUROTRAIN consortium to determine DNA methylation profiles and histone modification changes, and (iv) investigation of blood samples from patients with GTS for whole genome DNA methylation profiling (Zilhão et al., 2015), as mentioned in project 3.

### Project 5 integrated genetic networks underlying comorbid GTS and OCD (Joanna Widomska, Jan Buitelaar, Geert Poelmans, and Jeffrey Glennon, Radboud University Medical Center, Nijmegen)

The aim of this project is to determine the extent of “genetic overlap” in terms of shared underlying gene pathways and molecular signaling cascades between GTS and OCD and to provide further insights into how aberrant processes underlie these genetically related, clinically overlapping but still distinct neurodevelopmental disorders. Combining literature search approaches with diverse bioinformatics analytic tools (e.g., Ingenuity Pathway Analysis), top candidate genes emerging from GWASs of GTS (Scharf et al., 2013), OCD (Stewart et al., 2013; Mattheisen et al., 2015), and corroborating genetic evidence including data from recurrent and “genome-wide” CNV studies, candidate gene studies, miRNA expression data, animal models, and gene expression studies are selected and evaluated. The genes presenting overlap between GTS and OCD are ranked and used to construct integrated genetic networks that represent the “molecular landscape” of the overlapping traits between GTS and OCD, as well as GTS itself. The molecular landscape of OCD alone has recently been published (van de Vondervoort et al., 2016). This approach will be instrumental to discover unknown causative genes, pathways, and mechanisms and identify common pleiotropic genetic risk variants as possible therapeutic targets.

### Project 6 the genetic epidemiology of GTS, tics and related phenotypes (Nuno Rodrigues Zilhäo Nogueira, Dorret i. Boomsma and Danielle Cath, Utrecht University and Vu University Medical Center)

This study uses data that has been gathered by the NTR over the last 25 years, on twins, and family members (*n* = 16,896 individuals with SNP, epigenetic and expression data in subsamples), including a range of phenotypic data from questionnaires and genetic data. Structure equation model fitting procedures are used to model the phenotypic resemblance between family members and the relative contribution of genetic and environmental factors to variation and covariation among traits. Also, genome-wide association methodologies are being used to disentangle the genetic architecture underlying the etiology of GTS traits by estimating SNP heritability and polygenic risk scores for example.

### Project 7 developing algorithmic prediction models for GTS and related disorders (John Alexander and Peristera Paschou, Democritus University of Thrace)

With the continuous development of state of the art technologies for generating large amounts of genomic data, there is a need to develop new methodologies in order to identify promising SNPs, and candidate genes for further experimental validation. Using genetic data available for GTS and related disorders, this project develops, and applies new methodologies to scan high throughput genomic data (Genome Wide Association data, next generation sequence data, and microarrays). For example, using meta-analysis data comprised of 1285 GTS cases, and 4964 controls ancestry-matched to the GTS sample from the first GWAS (Scharf et al., 2013), we perform pathway, protein-protein interaction and gene-ontology analysis in order to dissect the molecular mechanisms underlying GTS. Furthermore, using novel bioinformatics tools for SNP based and gene based functional analysis, we perform candidate gene prioritization, gene set enrichment, and tissue enrichment analysis. We also construct functional interaction networks using combined information from the enriched functional and pathway results. This project will aid in highlighting pathways involved in the susceptibility of GTS and will bring out susceptibility factors that interact in order to confer risk for GTS.

## Animal models

Animal models of disease are an integral part of disease investigation and drug testing. However, ill-suited or inappropriate models are often used for these purposes. While multiple useful animal models for tic disorders exist, not all of these adequately mimic the syndrome, and crucially there is a lack of a juvenile model for GTS, despite it being a childhood onset disorder. Two animal model projects within TS-EUROTRAIN work to remedy these shortcomings, by developing a new juvenile GTS model within which the cortico-striato-thalamo-cortical (CSTC) circuitry and in particular the role of the glutamatergic system are being investigated. Furthermore, the effect of older and newer psychotropic compounds (e.g., riluzole and aripiprazole) are tested and novel targets identified. Similarly to the genetics and human neuroimaging projects a wide field of investigation is taken to include common comorbidities. Furthermore, samples from these projects undergo epigenetic testing as mentioned in project 4.

### Project 8 finding developmental aspects and possible drug targets of GTS and OCD: metabotropic glutamatergic mechanisms in a neurodevelopmental rat model of repetitive behaviors (Ester Nespoli and Bastian Hengerer, Boehringer Ingelheim pharma GmBH and Co. KG)

The unilaterally lesioned 6-hydroxidopamine (6-OHDA) adult rat is a well-established model used in Levodopa-induced Dyskinesia research. In this model a rapid degeneration of nigrostriatal neurons is chemically induced by the intrastriatal or intranigral administration of 6-OHDA, which selectively targets monoaminergic neurons. Chronic application of L-dopa to 6-OHDA lesioned rats leads to the development of repetitive involuntary movements, mainly involving the forepaw, neck, and mouth (Cenci et al., 1998). This appears as a consequence of the striatal super sensitivity to dopamine, caused by higher surface expression of dopamine receptors, which is a putative pathological mechanism of GTS and is induced in this model via previous dopamine deprivation (Buse et al., 2013). Here this model is translated to juvenile rats, inducing the lesion in postnatal days and monitoring its neurodevelopmental consequences. This provides new insights into the pathological mechanism of tics during development and a new tool to test therapeutic options for this disease.

### Project 9 investigation of the effect of classical and new psychotherapeutic approach in a rat model for GTS—a magnetic resonance spectroscopy (MRS) study (Francesca Rizzo and Andrea Ludolph, University of ULM)

This study compares the *in vivo* efficacy of a classical and a new therapeutic approach on tic management and their respective neurochemical effect in a rat model of GTS (Bronfeld et al., 2013). Aripiprazole is a second generation antipsychotic drug (classical approach) that has been found to be effective on tic management and to have a well-tolerated side effect profile (Kawohl et al., 2009). It is known that dopamine metabolism is dysfunctional in GTS, but neuroimaging research, and genetic studies also implicate other neurotransmitters in tic generation: histamine, serotonin, noradrenaline, endocannabinoids, glutamate, and GABA (Buse et al., 2013; Udvardi et al., 2013). Since the glutamate and dopamine systems are closely connected, a newly proposed approach for GTS treatment consists of the glutamatergic modulator riluzole, which is known to exert neuroprotection from glutamate excito-toxicity both *in vitro* and *in vivo* (Risterucci et al., 2006). Magnetic resonance spectroscopy (MRS) is used in an animal model to longitudinally analyse glutamate metabolites in the brain over a critical period of time in GTS; childhood through to early adulthood when tics appear and reach their maximum severity. The discovery of new pharmacological targets can provide new direction in drug development for GTS.

## Neuroimaging

**Figure 2:**
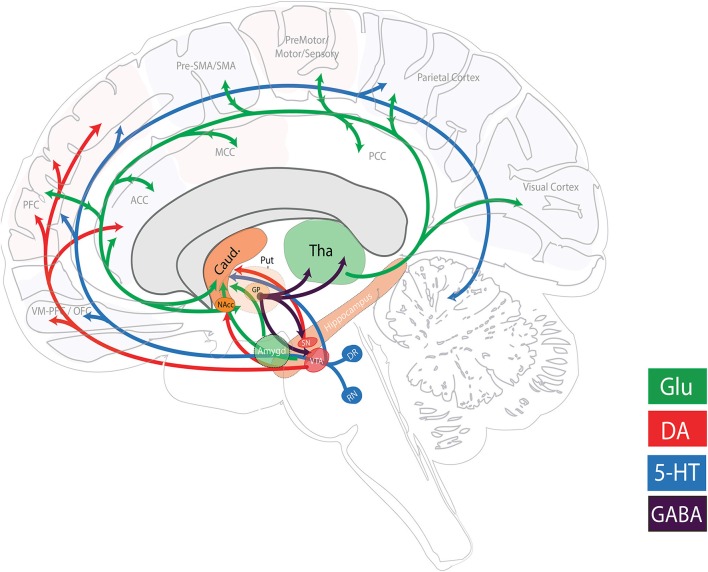
**Major neurotransmitter pathways related to GTS pathophysiology**. Simplified schematic illustration of the major neurotransmitter systems reported and hypothesized to be involved in GTS pathophysiology. Other neuromodulatory systems that have been implicated include the cholinergic, histaminergic, and endocannabinoid systems. The figure was adapted based on information from Singer (2013) and Schumann et al. (2010). (5-HT, serotonergic; ACC, anterior cingulate cortex; Amygd, amygdala; Caud, Caudate nucleus; DA, dopaminergic; DR, dorsal raphe nucleus; GABA, gamma-aminobutyric acid; Glu, glutamatergic; GP, globus pallidus; MCC, mid cingulate cortex; NAcc, nucleus accumbens; OFC, orbitofrontal cortex; PCC, posterior cingulate cortex; PFC, prefrontal cortex; Put, putamen; RN, raphe nucleus; SMA, supplementary motor area; SN, substantia nigra; Tha, thalamus; VM-PFC, ventromedial prefrontal cortex; VTA: ventral tegmental area).

Our three (human) neuroimaging projects are highly complementary with similar techniques used across all sites so as to allow for the cross-comparison of findings with limited methodological confounding factors. Projects 11 and 12 even pool data for certain comparisons. Each project utilizes MRS to evaluate the role of the glutamatergic system; T1-weighted structural magnetic resonance imaging (MRI) to examine structural brain differences; functional MRI (fMRI; resting state and task specific) data to interrogate the functional coupling between cognitive, limbic, and sensory-motor CSTC networks; and diffusion-weighted MRI (dMRI) data to inspect the structural connectivity. Each project does, however, differ in the populations under investigation and aims to address different unknown areas regarding GTS neurobiology. Together these works, along with the animal MRI study, may have implications on future glutamatergic modulatory therapies for tic suppression and could potentially extend the current pathophysiological model of GTS and related circuits beyond CSTC circuitry (Figure 2).

### Project 10 structural and functional neural correlates of pediatric GTS and ADHD (Natalie Forde, Jan Buitelaar, and Pieter Hoekstra, University Medical Center Groningen)

Few neuroimaging studies of GTS have investigated brain structure and function in children with even fewer longitudinal studies tracking the development of GTS (Ganos et al., 2013). Furthermore, the similarities and differences between ADHD and GTS have yet to be explicitly tested (Plessen et al., 2007). For this study structural, functional (resting state and task-dependent stop-signal and reward tasks) and dMRI data are acquired alongside MRS for glutamate and glutamine concentrations, neuropsychological, and phenotypic data from 180 children between 8–12 years of age (60 GTS with or without ADHD, 60 ADHD only, and 60 healthy controls). Common and unique neural correlates of GTS and ADHD are elucidated. Furthermore genetic data is acquired and will be analyzed as part of the EU-funded TACTICs project. Lastly a 3 year follow-up has been granted where the same battery of tests, including MRI, will be undertaken to allow the course of GTS and ADHD to be investigated.

### Project 11 studying the role of glutamate in CSTC circuit function and structure in adult GTS and OCD (Siyan Fan, Dick Veltman, Odile Van Den Heuvel, Petra Pouwels, Ysbrand Van Der Werf, and Danielle Cath, Department of Clinical and Health Psychology, Utrecht University and Vu University Medical Center)

The neural correlates of GTS and OCD have scarcely been compared and contrasted despite the high rate of co-occurrence (Freeman et al., 2000). This project is to investigate how altered glutamatergic function (as measured with MRS) is related to changes in structure (T1- and diffusion- weighted) and function (resting state and task-dependent stop-signal task) of the CSTC circuits in adult patients with GTS and OCD in comparison to healthy individuals. A similar range of neuroimaging, neuropsychological and phenotypic data to the above is acquired from adults with GTS, OCD and healthy controls (*n* = 20 per group). The participants with OCD as well as the controls have been chosen from a previous local OCD study while those with GTS are newly recruited. Genetic data is collected to contribute to genetic analysis within other projects of the network and to perform imaging-genetic analyses.

### Project 12 elemental, neurochemical, and network based analysis of the pathophysiological mechanisms of GTS (Ahmad Seif Kanaan, Harald Möller, and Kirsten Müller-Vahl, Hannover Medical School and Max Planck Institute For Human Cognitive And Brain Sciences)

Neuroimaging and behavioral data are acquired from up to 40 adult patients before and after treatment with the pharmacological agent aripiprazole, an atypical antipsychotic agent which is commonly used to treat GTS. At the elemental level, we use Quantitative Susceptibility Mapping (QSM) techniques to investigate whether patients exhibit an altered distribution of iron concentrations within basal ganglia nuclei in comparison to 40 healthy controls. At the neurochemical level, we investigate the role of the glutamatergic system within cortico-striatal regions using MRS at baseline and following treatment. At the network level, we use resting-state fMRI to investigate the interaction between large scale networks and their relationship to clinical status.

## Anticipated outcomes of TS-EUROTRAIN

TS-EUROTRAIN is a showcase of the potential impact of large-scale interdisciplinary and collaborative efforts aiming to understand GTS. Our basic science research combined with clinical neuroimaging studies will greatly increase our knowledge of the biological underpinnings of GTS and related disorders and allow a suitable biological model for these disorders to be established. The benefits of our research will include the potential identification of novel treatment targets and the availability of a suitable animal model on which to test newly developed pharmacotherapies targeting these newly identified biological pathways. This will ultimately lead to improved treatments and consequently increased quality of life for those suffering from GTS and their families. Despite being common, GTS is still considered a rare, unusual disease by the public, and has been associated with symptoms and signs causing social misunderstanding and stigmatization (Roessner et al., 2011; Robertson, 2015a). Undertaking a comprehensive scientific and outreach programme TS-EUROTRAIN has the important aspiration to help raise awareness about GTS, alleviate stigmatization, and transform GTS into a model disorder for the development of European policies for the promotion of childhood mental health.

## Author contributions

NF and AK are Joint first authors AL deceased NF and AK wrote the MS. JW, SP, EN, and JA generated/contributed to the figures and contributed to writing along with JR, SF, RH, MN, NZ, LP, and FR. PH and PP reviewed and supervised the writing this MS. TA, CB, TB, DB, JB, DC, AD, PD, JG, BH, OV, CJ, HM, KM, GP, PJWP, JS, HS, ZT, DV, YV, PH, and PP all have roles in setting up and/or supervising the individual projects described in the MS. MD, and WB represent the patient organizations involved in the establishment of the network. ND, SG, and TO also work on the projects.

## Funding

This network has been funded by the European Union under the Seventh Framework People Programme (TS-EUROTRAIN (FP7-PEOPLE-2012-ITN), Grant Agr.No.316978); we further acknowledge support by the European Union Seventh Framework Programmes [TACTICS (FP7/2007-2013), Grant Agr.No. 278948 and EMTICS (FP7/2007-2013), Grant Agr.No. 278367]. CB was supported by the Merit-prize scholarship of Semmelweis University and the János Bolyai Research Scholarship of the Hungarian Academy of Sciences BO/00987/16/5.

### Conflict of interest statement

JB has been in the past 3 years a consultant to / member of advisory board of / and/or speaker for Janssen Cilag BV, Eli Lilly, Medice, Shire, Lundbeck, Roche and Servier. He is neither an employee of any of these companies nor a stock shareholder of any of these companies. He has no other financial or material support, including expert testimony, patents, or royalties. PH has been in the past three years consultant to / member of advisory board of Shire. OV received speakers' honorarium from Lundbeck and received research funding from PhotoPharmics. The other authors declare that the research was conducted in the absence of any commercial or financial relationships that could be construed as a potential conflict of interest.
